# Supplementary material for: In Vitro Validation of Phosphorodiamidate Morpholino Oligomers
Source: Molecules. 2019 Aug 12;24(16):2922. doi: 10.3390/molecules24162922 (PMC6719133; doi:10.3390/molecules24162922)
Supplement: Supplementary file 1 [file molecules-24-02922-s001.pdf]

molecules

In Vitro Validation of Phosphorodiamidate Morpholino Oligomers

May T. Aung-Htut [1,2,†], Craig S. McIntosh [1,2,*,†], Kristin A. West [1], Sue Fletcher [1,2] and Steve D. Wilton [1,2]

[1] Centre for Molecular Medicine and Innovative Therapeutics, Murdoch University, Perth 6150, Western Australia, Australia
[2] Perron Institute for Neurological and Translational Science, The University of Western Australia, Perth 6009, Western Australia, Australia
* Correspondence: c.mcintosh@murdoch.edu.au
† These authors contributed equally to this work.

Academic Editor: Rakesh N. Veedu
Received: 19 June 2019; Accepted: 8 August 2019; Published: date

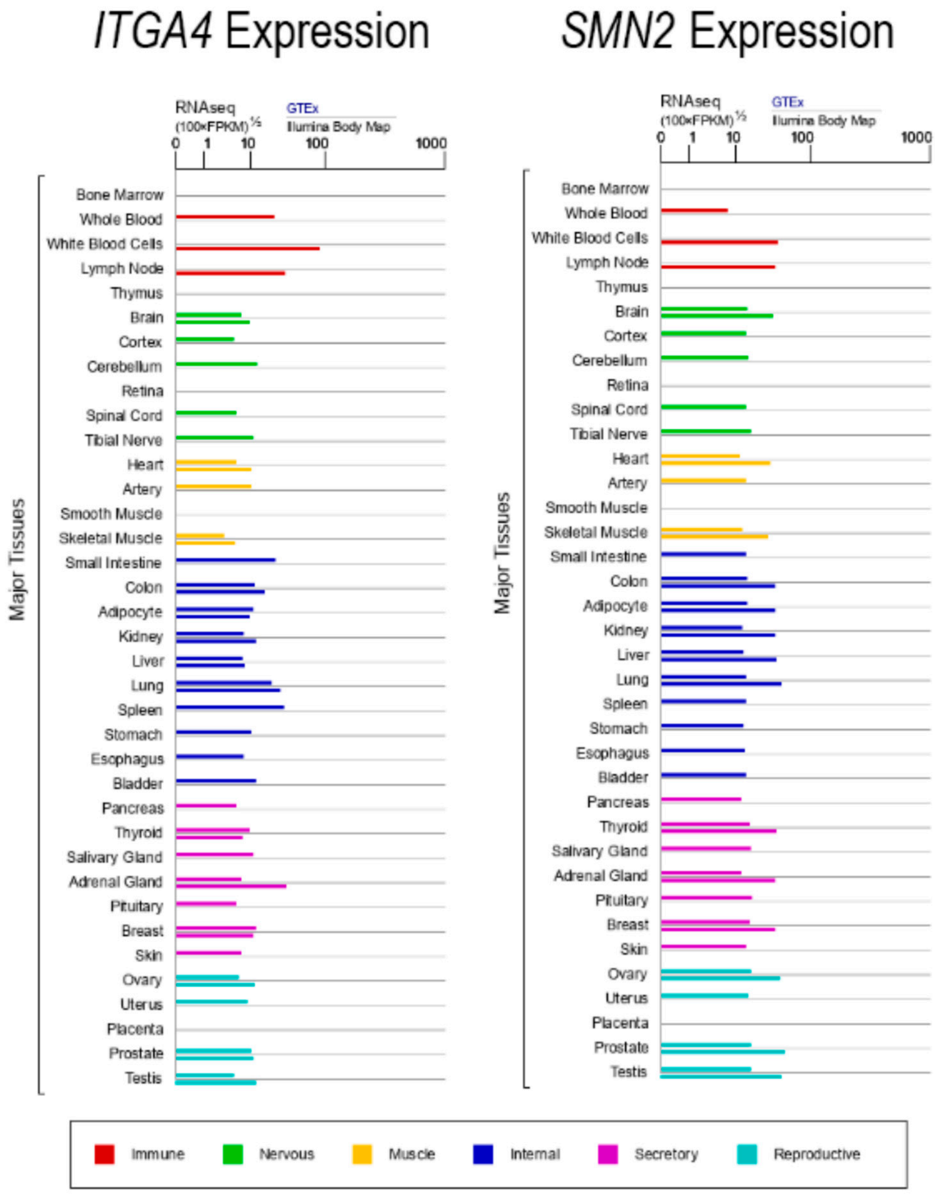

Figure S1. Genecards.org gene expression profile of human *ITGA4* and *SMN2* transcripts in major tissues. Expression values calculated through RNAseq. Schematics of expression adapted from information located at www.genecards.org.

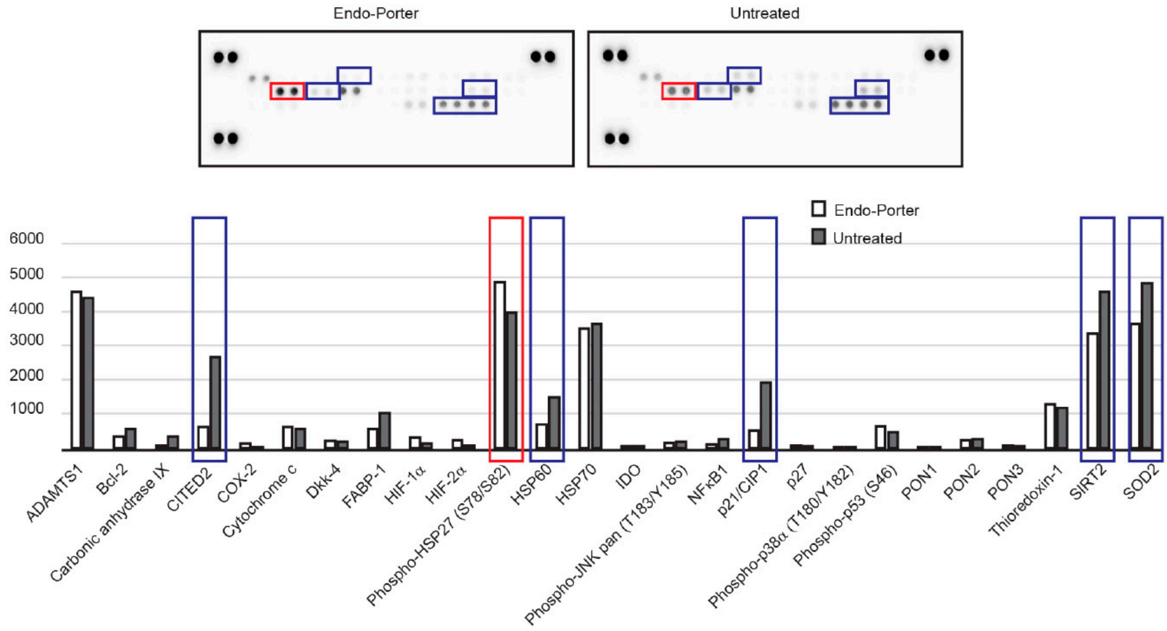

Figure S2. Normal human dermal fibroblasts were either untreated or treated with Endo-Porter for 48 hours and 200 μg of cell lysates were analysed on human stress arrays. The graph shows the pixel density of protein blots analysed by densitometric image analysis (image J) in the order from top left to bottom right excluding the three positive controls pairs (top and bottom left and top right corners of the membrane).

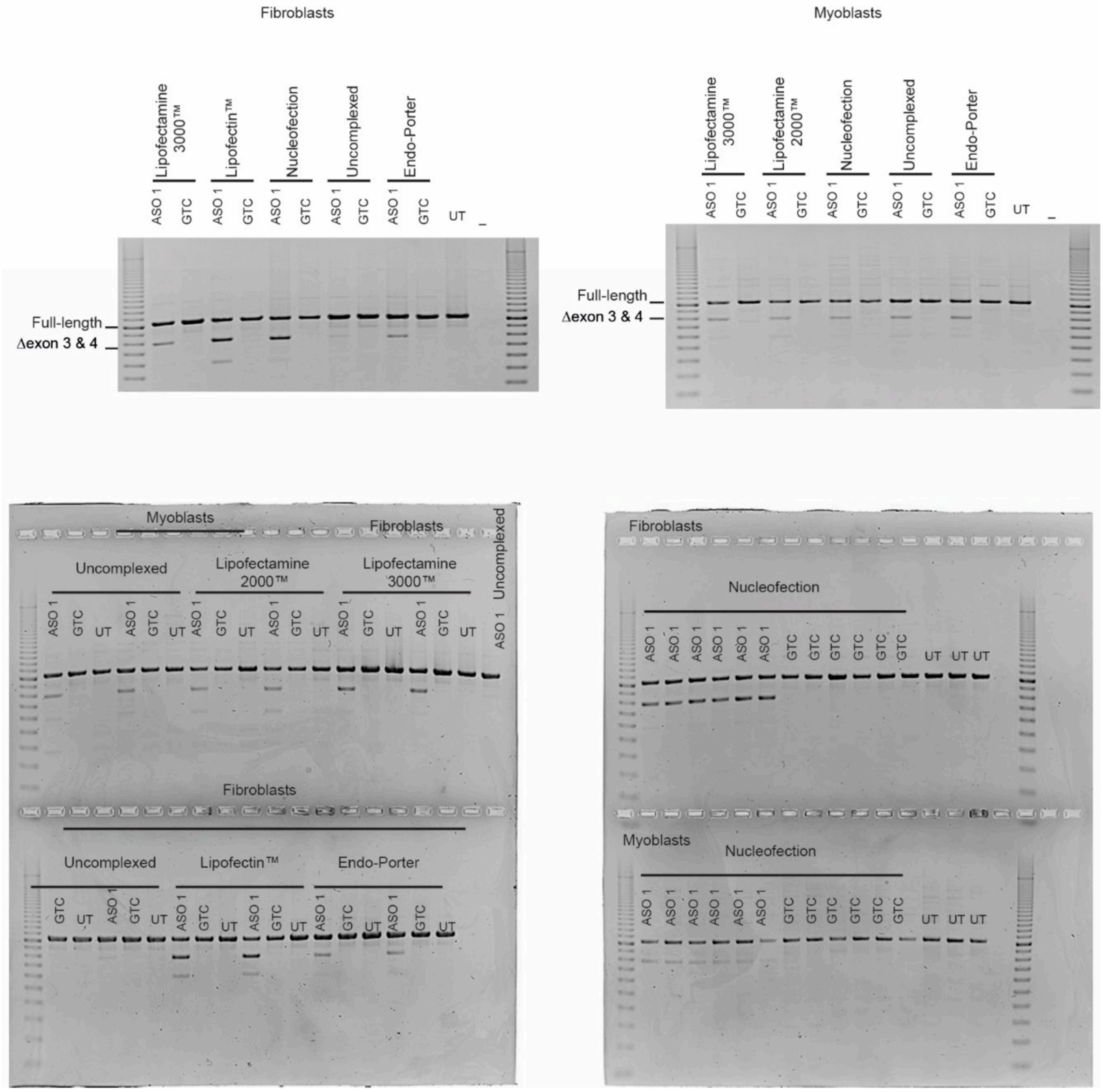

Figure S3. Experimental biological replicates for Figure 1.
